# Impact of antiviral therapy on short‐ and long‐term outcomes of patients with chronic obstructive pulmonary disease after influenza infection

**DOI:** 10.1111/irv.13231

**Published:** 2023-12-13

**Authors:** Christopher Wallick, Tu My To, Stephan Korom, Henry Masters, Ning Wu, Dalia Moawad, Nicola A. Hanania

**Affiliations:** ^1^ Genentech, Inc. South San Francisco California USA; ^2^ F. Hoffmann‐La Roche Ltd. Basel Switzerland; ^3^ Section of Pulmonary, Critical Care and Sleep Medicine Baylor College of Medicine Houston Texas USA

**Keywords:** antivirals, chronic obstructive pulmonary disease, costs, healthcare resource utilization, influenza, long‐term

## Abstract

**Background:**

Respiratory complications often accompany influenza in patients with chronic obstructive pulmonary disease (COPD). In this retrospective study, we quantified the impact of antiviral therapy on exacerbations, healthcare resource utilization (HRU), and costs in patients with COPD across 5 influenza seasons.

**Methods:**

Using claims data from US MarketScan® databases, we identified patients with COPD who had an influenza diagnosis during the 2012–2016 influenza seasons. Patients who received a neuraminidase inhibitor within 48 h of diagnosis (*N* = 4134) were identified and propensity score–matched 1:1 to a comparator cohort of untreated patients. We determined COPD‐ and pneumonia‐related HRU and costs during month 1, each subsequent quarter, and months 2–13.

**Results:**

Antiviral‐treated patients had a significantly lower frequency of COPD‐related outcomes than untreated patients during all periods (exacerbations: 10.4% vs 18.2% [month 1] and 17.7% vs 24.2% [months 2–13]; inpatient visit: 2.5% vs 7.9% [month 1] and 3.8% vs 6.7% [months 2–13]; *P* < 0.0001, all comparisons). Treated patients also had significantly lower outpatient and emergency department (ED) visits beyond month 1. Pneumonia‐related inpatient, ED, and outpatient visits were significantly lower in antiviral‐treated patients than in untreated patients over all periods (*P* < 0.0001, all comparisons). In all HRU categories, COPD‐ and pneumonia‐related costs were significantly lower in treated patients over all periods (month‐1 ED visit costs were higher).

**Conclusions:**

Antiviral treatment in patients with COPD and influenza is associated with significantly lower HRU and costs in the postinfection month and for an entire year following infection compared with untreated patients.

## 1 INTRODUCTION

Chronic obstructive pulmonary disease (COPD) is a chronic inflammatory lung disease associated with airway obstruction that results in respiratory symptoms such as difficulty in breathing, shortness of breath, wheezing, and chest tightness. COPD is estimated to affect 24 million people in the United States and is the third leading cause of death after heart disease and cancer.^1^ The economic burden of COPD has increased from $32.1 billion in 2010 to a projected $49 billion in 2020.^2^ The chronic and progressive course of COPD is often punctuated by exacerbations, defined clinically as episodes of worsening respiratory symptoms, particularly increased dyspnea, cough, and sputum purulence as well as lower oxygen levels.^3^ COPD exacerbations accelerate disease progression and are associated with worsening respiratory function, reduced quality of life, increased risk of hospitalizations, and higher healthcare costs.^4^, ^5^, ^6^, ^7^

Respiratory viruses are a common trigger of COPD exacerbations, and influenza virus is one of the three most common respiratory viruses detected in patients with an exacerbation of COPD.^8^, ^9^, ^10^, ^11^ Influenza is associated with overproduction of cytokines and chemokines that can lead to severe inflammation, including excessive recruitment of neutrophils and mononuclear cells at the site of infection.^12^, ^13^ Exacerbations associated with viral infections are likely to be more severe and are associated with greater decline in pulmonary function and longer hospitalization.^14^ Additionally, secondary bacterial infections stemming from disruption of the respiratory tract by influenza virus often result in a synergistic decline of lung function and worse outcomes.^15^, ^16^, ^17^, ^18^ Acute pneumonia has been diagnosed in 30% to 40% of hospitalized patients with influenza, particularly patients with risk factors such as chronic heart or lung disease and a history of smoking.^19^ In patients with COPD, influenza can lead to an exacerbation, and viral pneumonia or secondary bacterial pneumonia is often the cause of hospitalization or death. However, even after the resolution of influenza infection, the respiratory health of COPD patients continues to deteriorate for at least a year after the acute episode.^20^

Because of the significant contribution of influenza virus to COPD exacerbations, annual influenza vaccination is recommended for patients with COPD, but vaccination coverage rates in this population remain suboptimal.^21^, ^22^ Early treatment with antivirals reduces symptoms and complications in the general population^23^, ^24^ but rates of antiviral prescribing among patients with acute respiratory illness in both hospital and outpatient settings are also low.^21^, ^25^, ^26^

In view of the high health and cost burden of influenza in patients with COPD and to guide clinical decision making, data on the effect of antivirals on complications and other healthcare outcomes in COPD patients specifically are needed. In this observational retrospective cohort study, we used real‐world US claims data from five influenza seasons in the United States (2012 through 2016) to examine the effect of antiviral agents on influenza‐related complications, healthcare resource utilization (HRU), and costs in COPD patients with influenza.

## 2 METHODS

### 2.1 Data source

**FIGURE 1:**
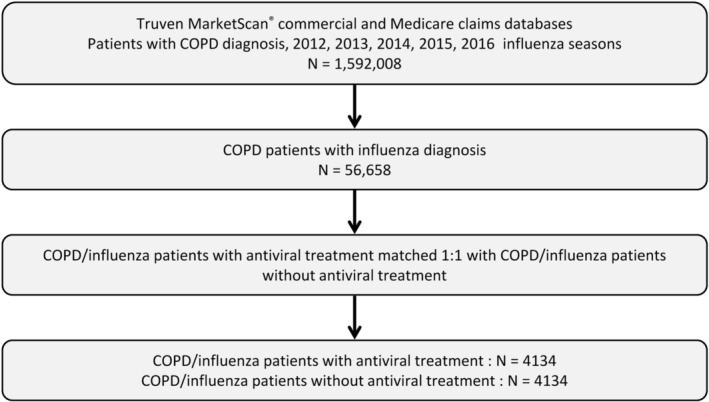
Study design. COPD, chronic obstructive pulmonary disease.

Data for this retrospective cohort study were extracted from the IBM MarketScan® Commercial Claims and Encounters Database and the MarketScan® Medicare Supplemental and Coordination of Benefits Database (IBM Watson Health, Cambridge, MA) (Figure 1). The claims files capture inpatient and outpatient care, use of facilities and services, prescription fills, and payment information. Each medical claim had up to 15 diagnosis codes (ICD‐9‐CM [International Classification of Diseases, 9th Revision, Clinical Modification], ICD‐10) and 15 procedure codes (ICD PCS [Procedure Coding System], CPT [Current Procedural Terminology], and HCPCS [Healthcare Common Procedure Coding System]) documented for billing purposes. The study used deidentified data and was exempt from Institutional Review Board review. The research was compliant with the Health Insurance Portability and Accountability Act.

### 2.2 Study cohorts

Patients with COPD were identified in 2‐year time frames that included the influenza season and the preceding 1‐year period. Patients were included if they were ≥40 years old, had ≥1 inpatient admission with a primary diagnosis of COPD, or had ≥2 outpatient visits for COPD dated at least 30 days apart and at least 2 non‐oral steroid COPD prescription fills within 1 year. The ICD‐9 or ICD‐10 diagnosis codes for identifying COPD patients were chronic bronchitis (491, J41, J42), emphysema (491, J43, J98), chronic airway obstruction (496), or COPD (J44). Prescription medications used to qualify COPD cases for inclusion in the study included bronchodilators, steroids, and phosphodiesterase (PDE)/PDE4 inhibitors.

Cases of influenza among patients with COPD were identified for five influenza seasons (2012 through 2016), with each season running from October 1 of each year to September 30 of the successive year. Patients were included if they had a claim associated with a diagnosis suggesting influenza (ICD‐9 codes 487.xx or 488.xx; ICD‐10 codes J09.xx, J10.xx, or J11.xx). For each patient, only the first influenza episode of the season was included, with the index date being the date of diagnosis. To be included, patients had to be continuously enrolled for at least 12 months before and 13 months after the index date.

The treated cohort consisted of patients who had a claim for an antiviral agent (oseltamivir, zanamivir, rimantadine, or peramivir) within 48 h of the index diagnosis. The untreated cohort consisted of COPD patients with influenza who were propensity score–matched 1:1 (nearest neighbor matching) to treated patients. Propensity scores were derived from logistic regression models that included the following variables in each influenza season: age at influenza diagnosis, sex, geographic region, type of health plan, month of index event, and Charlson comorbidity index (CCI) as well as measures of COPD severity (COPD treatment ratio [CTR; ratio of controller (maintenance) medications to total COPD medication],^27^ number of baseline COPD‐related outpatient visits, and number of baseline COPD‐related hospitalizations).

### 2.3 Outcomes

Outcomes were assessed during the 1‐month acute phase after influenza diagnosis/index infection as well as over the full year after the initial 1‐month period (months 2–13 combined after the index date). Outcomes for the 1‐year period after the 1‐month acute phase were additionally assessed quarterly (each quarter was a 3‐month period).

The percentage of patients with exacerbation of COPD or any COPD‐ or pneumonia‐related outcome was determined based on relevant ICD‐9 or ICD‐10 codes. COPD exacerbation was defined as a hospitalization for COPD or receipt of an oral steroid prescription within 2 weeks of a COPD‐related outpatient visit. Pneumonia was identified with the following codes: viral/bacterial/unspecified pneumonia (codes 480–486, 487.0, J12–J18); influenza due to certain identified influenza viruses with pneumonia (488.01, 488.11, 488.81), influenza due to identified novel influenza A virus with pneumonia (J09.X1), influenza due to other identified influenza virus with pneumonia (J10.0), and influenza due to unidentified influenza virus with pneumonia (J11.0). COPD‐ and pneumonia‐related HRU and costs were also assessed. These included the length of inpatient stay and the number and total cost of inpatient, emergency department (ED), and outpatient visits. Costs were adjusted to 2018 US dollars using the medical component of the Consumer Price Index.

### 2.4 Statistical analysis

Outcomes of COPD patients with influenza who received antiviral treatment were compared with those in untreated patients for each period (month 1 after diagnosis, quarter 1 [Q1; months 2–4], Q2 [months 5–7], Q3 [months 8–10], Q4 [months 11–13], and months 2–13 combined) with the chi‐squared test for categorical measures and Wilcoxon signed‐rank test for counts and costs. Analyses were conducted using SAS v9.4 software (SAS Institute Inc., Cary, NC).

## 3 RESULTS

### 3.1 Study population

Of 1,592,000 patients diagnosed with COPD over the 2012 through 2016 influenza seasons, 56,658 patients had a claim for an influenza diagnosis. Following propensity score matching, a treated and an untreated cohort, each with 4134 patients with COPD, was identified for the 5‐season study period (Figure 1).

Treated and untreated cohorts were generally well matched for the listed variables. Patients 65 years and older comprised 39.6% of the treated group and 48.2% of the untreated group. The CCI was ≥2 in 37.6% of the treated group and 44.3% of the untreated group. A CCI of 0 was reported in approximately 6% of patients; this was possible because the CCI was determined in the 1‐year baseline period preceding the index date, and some patients may not have had COPD‐related visits or medication in this period. A slightly lower percentage of treated patients than untreated patients had ≥6 COPD‐related outpatient visits (22.4% vs 28.8%) or had a COPD‐related hospitalization (4.6% vs 6.4%) at baseline (Table 1).

**TABLE 1:** Baseline demographic and clinical characteristics of untreated and treated COPD patients with influenza during the 2012 through 2016 influenza seasons.

	No antiviral treatment (*N* = 4134) *n* (%)	Antiviral treatment (*N* = 4134) *n* (%)
Influenza season		
2012	748 (18.1)	748 (18.1)
2013	622 (15.1)	622 (15.1)
2014	1380 (33.4)	1380 (33.4)
2015	604 (14.6)	604 (14.6)
2016	780 (18.9)	780 (18.9)
Month of index event		
December–March	3551 (85.9)	3551 (85.9)
April–September	349 (8.4)	349 (8.4)
October–November	234 (5.7)	234 (5.7)
Age (year)		
<65	2139 (51.7)	2495 (60.4)
65–74	816 (19.7)	733 (17.7)
75+	1179 (28.5)	906 (21.9)
Sex		
Female	2445 (59.1)	2415 (58.4)
Male	1689 (40.9)	1719 (41.6)
Region		
North Central	1252 (30.3)	1038 (25.1)
Northeast	857 (20.7)	732 (17.7)
South	1708 (41.3)	2074 (50.2)
West	16 (0.4)	20 (0.5)
Unknown	301 (7.3)	270 (6.5)
Plan Type		
HMO	315 (7.6)	325 (7.9)
PPO	1498 (36.2)	1349 (32.6)
Other	2270 (54.9)	2418 (58.5)
Unknown	51 (1.2)	42 (1.0)
Charlson comorbidity index		
0	231 (5.6)	257 (6.2)
1	2071 (50.1)	2324 (56.2)
2	427 (10.3)	366 (8.9)
3+	1405 (34.0)	1187 (28.7)
CTR		
0	493 (11.9)	469 (11.3)
<0.2	45 (1.1)	59 (1.4)
0.2–0.4	175 (4.2)	183 (4.4)
0.4–0.6	554 (13.4)	486 (11.8)
0.6–0.8	901 (21.8)	869 (21.0)
>0.8	1966 (47.6)	2068 (50.0)
No. of baseline COPD‐related outpatient visits		
0	604 (14.6)	620 (15.0)
1–5	2344 (56.7)	2591 (62.7)
6–10	503 (12.2)	405 (9.8)
11–20	453 (11.0)	363 (8.8)
>20	230 (5.6)	155 (3.8)
No. of baseline COPD‐related inpatient visits		
0	3868 (93.6)	3942 (95.4)
1	244 (5.9)	183 (4.4)
2+	22 (0.5)	9 (0.2)

Abbreviations: COPD, chronic obstructive pulmonary disease; CTR, ratio of controller (maintenance) medications to total COPD medications; ED, emergency department; HMO, health maintenance organization; PPO, preferred provider organization.

### 3.2 COPD‐related outcomes

**FIGURE 2:**
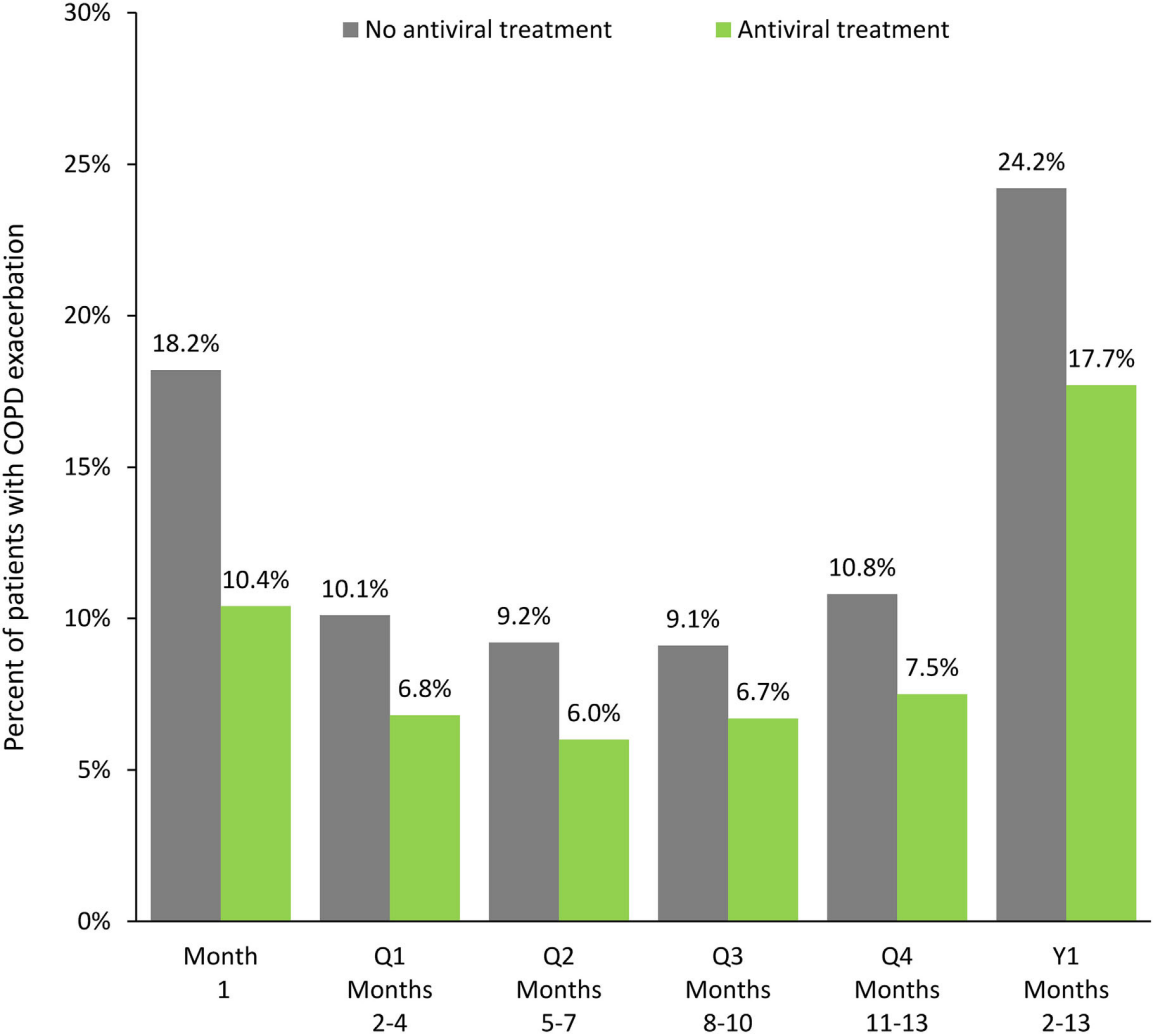
Proportion of patients with COPD exacerbation in treated and untreated cohorts during the 2012 through 2016 influenza seasons. *P <* 0.0001 for all comparisons (antiviral treatment vs no antiviral treatment). COPD, chronic obstructive pulmonary disease; Q, quarter; Y, year.

**FIGURE 3:**
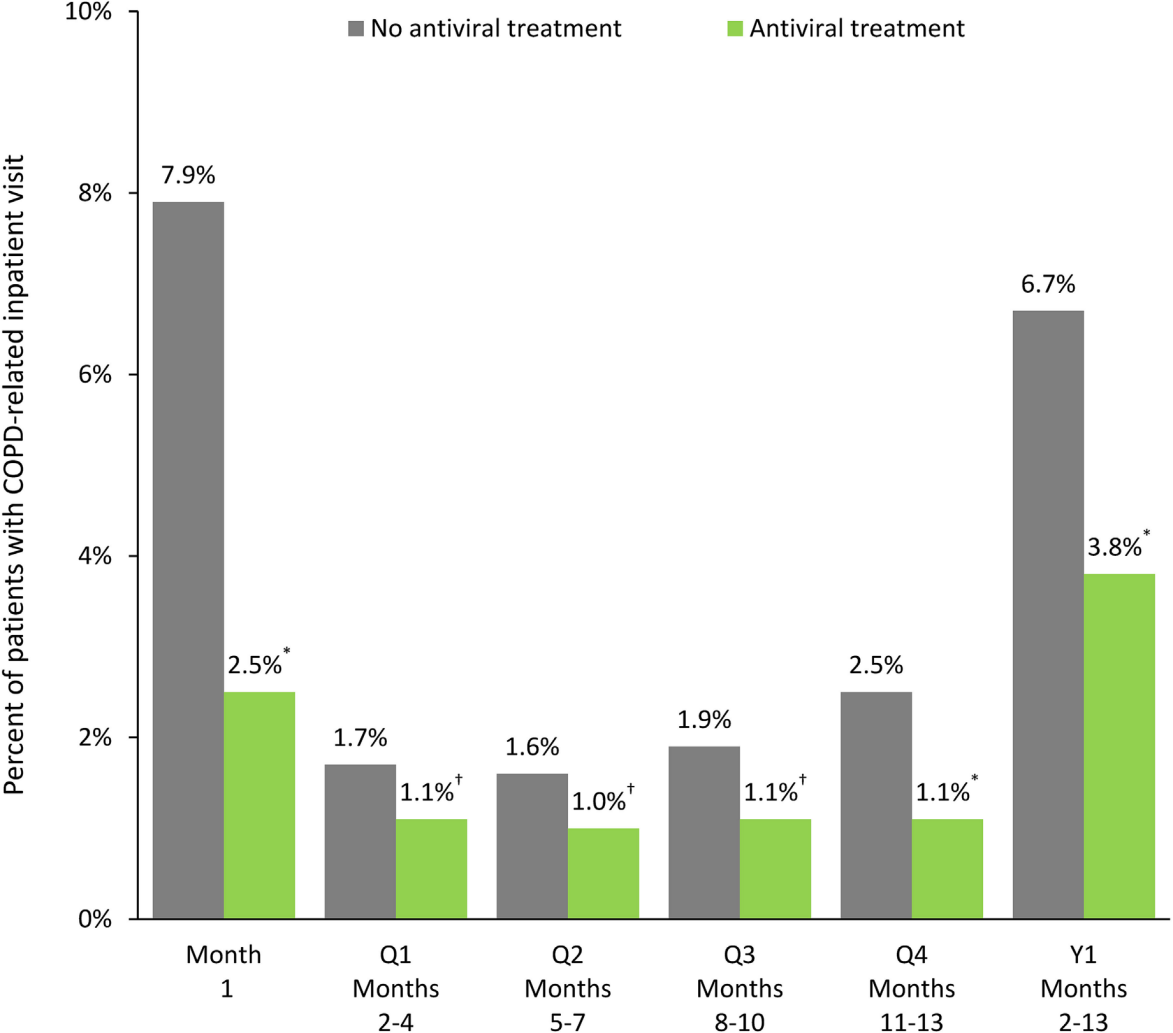
Proportion of patients with a COPD‐related inpatient visit in treated and untreated cohorts during the 2012 through 2016 influenza seasons. *P < 0.0001; ^†^ *P* < 0.05 (antiviral treatment vs no antiviral treatment). COPD, chronic obstructive pulmonary disease; Q, quarter; Y, year.

The proportion of patients with an exacerbation was significantly lower in antiviral‐treated patients than in untreated patients not only in the first month after infection (10.4% vs 18.2%; *P* < 0.0001) but also over the succeeding full year (17.7% vs 24.2%; *P* < 0.0001) and during each quarter after month 1 (*P* < 0.0001 for all comparisons) (Figure 2). Over each time interval studied, significantly fewer patients in the treated group than in the untreated group had a COPD‐related inpatient visit (month 1: 2.5% vs 7.9%, *P* < 0.0001; months 2–13: 3.8% vs 6.7%, *P* < 0.0001) (Figure 3) or outpatient visit (*P* < 0.0001 for all periods) (Table 2). The proportion of patients with an ED visit was also significantly lower among treated patients over all periods (*P* < 0.01 to *P* < 0.0001) with the exception of month 1 (*P* = 0.55) (Table 2).

**TABLE 2:** COPD‐ and pneumonia‐related outcomes over 13 months in treated and untreated cohorts during the 2012 through 2016 influenza seasons.

Antiviral treatment	Percent of patients with outcome											
	Month 1		Q1 (M2–M4)		Q2 (M5–M7)		Q3 (M8–M10)		Q4 (M11–M13)		Y1 (M2–M13)	
	No	Yes	No	Yes	No	Yes	No	Yes	No	Yes	No	Yes
COPD‐related outcome												
Inpatient visit	7.9%	2.5%^a^	1.7%	1.1%^b^	1.6%	1.0%^b^	1.9%	1.1%^a^	2.5%	1.1%^a^	6.7%	3.8%^a^
ED visit	11.5%	11.1%	6.5%	4.8%^a^	6.1%	4.1%^a^	6.3%	4.8%^c^	7.1%	5.6%^c^	19.7%	15.7%^a^
Outpatient visit	60.0%	48.0%^a^	62.6%	53.7%^a^	57.6%	50.0%^a^	57.0%	48.4%^a^	54.5%	49.1%^a^	85.3%	82.4%^a^
Exacerbations	18.2%	10.4%^a^	10.1%	6.8%^a^	9.2%	6.0%^a^	9.1%	6.7%^a^	10.8%	7.5%^a^	24.2%	17.7%^a^
Pneumonia‐related outcome												
Inpatient visit	28.7%	8.1%^a^	4.9%	2.8%^a^	4.2%	2.7%^a^	4.4%	2.4%^a^	5.5%	2.8%^a^	15.7%	9.2%^a^
ED visit	5.3%	3.2%^a^	2.2%	1.3%^c^	1.8%	1.0%^c^	1.7%	1.1%^b^	2.2%	1.4%^b^	7.1%	4.5%^a^
Outpatient visit	25.5%	12.0%^a^	14.6%	7.1%^a^	7.9%	4.5%^a^	7.5%	4.1%^a^	7.9%	4.7%^a^	24.4%	14.9%^a^

Abbreviations: COPD, chronic obstructive pulmonary disease; ED, emergency department; M, month; Q, quarter; Y, year.

^a^ *P* < 0.0001.

^b^ *P* < 0.05.

^c^ *P* < 0.01.

The number of COPD exacerbations per patient was significantly lower in the treated group than in the untreated group in the first month, the next full year, and all 4 quarters (*P* < 0.0001 for all comparisons). The duration of inpatient stay was significantly shorter and the number of inpatient, ED, and outpatient visits for COPD‐related complications were significantly lower in treated patients compared with untreated patients over each period (*P* < 0.05 to *P* < 0.001); the only exception was the number of ED visits during month 1, which was similar between the two groups (*P* = 0.55) (Table 3).

**TABLE 3:** COPD‐ and pneumonia‐related healthcare resource utilization in treated and untreated cohorts during the 2012 through 2016 influenza seasons.

Antiviral treatment	Mean no. of outcomes (SD)											
	Month 1		Q1 (M2–M4)		Q2 (M5–M7)		Q3 (M8–M10)		Q4 (M11–M13)		Y1 (M2–M13)	
	No	Yes	No	Yes	No	Yes	No	Yes	No	Yes	No	Yes
COPD‐related outcomes												
No. of days of inpatient stay	0.38 (1.64)	0.08 (0.61)^a^	0.08 (0.78)	0.04 (0.57)^b^	0.09 (0.77)	0.04 (0.45)^b^	0.1 (0.87)	0.05 (0.75)^c^	0.12 (0.92)	0.06 (0.73)^a^	0.39 (1.85)	0.19 (1.31)^a^
No. of inpatient visits	0.08 (0.28)	0.03 (0.16)^a^	0.02 (0.13)	0.01 (0.1)^b^	0.02 (0.13)	0.01 (0.1)^b^	0.02 (0.14)	0.01 (0.11)^c^	0.02 (0.16)	0.01 (0.11)^a^	0.08 (0.31)	0.04 (0.23)^a^
No. of outpatient visits	1.48 (2.27)	0.89 (1.43)^a^	2.34 (3.70)	1.54 (3.29)^a^	2.01 (3.26)	1.43 (2.98)^a^	1.95 (3.13)	1.40 (2.93)^a^	1.90 (3.24)	1.46 (3.05)^a^	8.2 (10.85)	5.83 (10.27)^a^
No. of ED visits	0.14 (0.43)	0.13 (0.41)^a^	0.09 (0.45)	0.06 (0.27)^d^	0.09 (0.44)	0.05 (0.29)^a^	0.09 (0.39)	0.06 (0.32)^c^	0.09 (0.37)	0.07 (0.34)^c^	0.35 (1.09)	0.24 (0.74)^a^
No. of exacerbations per patient	0.23 (0.54)	0.13 (0.41)^a^	0.15 (0.51)	0.10 (0.42)^a^	0.14 (0.52)	0.08 (0.37)^a^	0.14 (0.54)	0.10 (0.42)^a^	0.17 (0.56)	0.11 (0.44)^a^	0.59 (1.58)	0.38 (1.17)^a^
Pneumonia‐related outcomes												
No. of days of inpatient stay	1.53 (3.12)	0.28 (1.23)^a^	0.41 (2.74)	0.13 (1.01)^a^	0.24 (1.36)	0.13 (0.96)^a^	0.26 (1.57)	0.14 (1.31)^a^	0.34 (1.87)	0.20 (1.83)^a^	1.25 (4.61)	0.60 (3.15)^a^
No. of inpatient visits	0.29 (0.47)	0.08 (0.28)^a^	0.05 (0.25)	0.03 (0.18)^a^	0.05 (0.23)	0.03 (0.18)^a^	0.05 (0.23)	0.03 (0.17)^a^	0.06 (0.26)	0.03 (0.19)^a^	0.21 (0.58)	0.11 (0.4)^a^
No. of outpatient visits	0.68 (1.97)	0.22 (0.86)^a^	0.44 (1.90)	0.17 (1.07)^a^	0.27 (1.84)	0.12 (0.83)^a^	0.23 (1.75)	0.13 (1.22)^a^	0.21 (1.18)	0.11 (0.76)^a^	1.15 (5.19)	0.53 (2.45)^a^
No. of ED visits	0.06 (0.26)	0.03 (0.19)^a^	0.02 (0.18)	0.01 (0.12)^c^	0.02 (0.16)	0.01 (0.12)^c^	0.02 (0.15)	0.01 (0.14)^b^	0.02 (0.16)	0.02 (0.15)^b^	0.09 (0.35)	0.06 (0.29)^a^

Abbreviations: COPD, chronic obstructive pulmonary disease; ED, emergency department; M, month; Q, quarter; SD, standard deviation; Y, year.

^a^ *P* < 0.0001.

^b^ *P* < 0.05.

^c^ *P <* 0.01.

^d^ *P* < 0.001.

### 3.3 Pneumonia‐related outcomes

During month 1 and over the next full year, the proportion of patients with pneumonia‐related inpatient, ED, and outpatient visits was significantly lower in antiviral‐treated patients than in untreated patients (*P* < 0.0001 for all comparisons) (Table 2). During month 1, inpatient visits were 3‐fold lower (8.1% vs 28.7%) and outpatient visits 2‐fold lower (12.0% vs 25.5%), among treated patients than among untreated patients. For each quarter, the number of inpatient, ED, and outpatient visits was lower among treated patients than among untreated patients (*P* < 0.05 to *P* < 0.0001).

All categories of HRU, including duration of inpatient stay and number of inpatient, outpatient, and ED visits for pneumonia‐related complications, were significantly lower in treated patients than in untreated patients in the first month and the next full year (*P* < 0.0001 for all comparisons) (Table 3). Significant differences in all these outcomes between treated and untreated patients were also observed over all 4 quarters.

### 3.4 COPD‐ and pneumonia‐related healthcare costs

In untreated patients, the main COPD‐related cost drivers during month 1 were inpatient costs, but over the next full year, outpatient costs also increased (Table 4). By contrast, pneumonia‐related costs remained driven mainly by inpatient visits over all periods. Both COPD‐ and pneumonia‐related costs in all HRU categories in the first month and year (months 2–13) were significantly lower in the treated group than in untreated patients (*P* ≤ 0.0001); the exception was COPD‐related ED visit costs, which were higher among treated patients during month 1 (*P* = 0.75) (Table 4). Over the first month, costs for COPD‐related inpatient and outpatient visits were 4‐fold lower ($329 vs $1339) and 2.5‐fold lower ($177 vs $451), respectively, in treated patients than in untreated patients. Costs for pneumonia‐related inpatient and outpatient visits over the first month were ~5‐fold lower ($1199 vs $5856) and 7.3‐fold lower ($43 vs $315), respectively, in treated patients than in untreated patients. Treated patients continued to incur significantly lower costs than untreated patients for both COPD‐ and pneumonia‐related complications during each succeeding quarter.

**TABLE 4:** COPD‐ and pneumonia‐related healthcare costs in treated and untreated cohorts during the 2012 through 2016 influenza seasons.

Antiviral treatment	Mean costs, USD (SD)											
	Month 1		Q1 (M2–M4)		Q2 (M5–M7)		Q3 (M8–M10)		Q4 (M11–M13)		Y1 (M2–M13)	
	No	Yes	No	Yes	No	Yes	No	Yes	No	Yes	No	Yes
COPD‐related costs												
Inpatient	1339 (7907)	329 (2779)^a^	264 (2650)	149 (2374)^b^	568 (20042)	115 (1236)^b^	337 (3517)	250 (8826)^c^	522 (4587)	183 (2615)^a^	1690 (21170)	697 (9629)^a^
Outpatient	451 (1849)	177 (880)^a^	643 (2541)	437 (2226)^a^	539 (2852)	343 (1359)^a^	465 (1844)	346 (1545)^a^	511 (2091)	387 (1844)^a^	2158 (6169)	1513 (4762)^a^
Emergency department	363 (2290)	406 (2173)^a^	220 (1600)	180 (1743)^d^	266 (2242)	146 (1455)^a^	218 (1563)	195 (1875)^c^	308 (2251)	223 (1635)^c^	1012 (4491)	745 (3625)^a^
Pneumonia‐related costs												
Inpatient	5856 (16984)	1199 (5447)^a^	1421 (10091)	484 (3997)^a^	1155 (20581)	530 (5877)^a^	939 (6429)	642 (10514)^a^	1512 (10477)	599 (5599)^a^	5027 (27423)	2255 (15781)^a^
Outpatient	315 (2548)	43 (267)^a^	168 (1503)	52 (574)^a^	79 (795)	39 (598)^a^	56 (592)	43 (785)^a^	63 (740)	52 (1200)^a^	366 (2179)	186 (1828)^a^
Emergency department	133 (1372)	125 (1202)^a^	90 (1205)	54 (807)^c^	83 (1188)	44 (754)^c^	87 (1104)	79 (1187)^b^	120 (1653)	81 (1245)^b^	381 (2656)	258 (2054)^a^

Abbreviations: COPD, chronic obstructive pulmonary disease; M, month; Q, quarter; Y, year.

^a^ *P* < 0.0001.

^b^ *P* < 0.05.

^c^ *P <* 0.01.

^d^ *P* < 0.001.

## 4 DISCUSSION

Seasonal influenza adds to the substantial burden of disease in patients with COPD. Early antiviral treatment may attenuate the severity and frequency of exacerbations and decrease the associated health and cost burden, but the effect of these agents on the progression of COPD has not been well documented. In this real‐world study, we used claims data from 5 influenza seasons in the United States to investigate short‐ and long‐term effects of antiviral treatment on the frequency of COPD exacerbations and COPD‐ and pneumonia‐related HRU and costs. We demonstrate that COPD patients with influenza who received an antiviral within 2 days of influenza diagnosis had significantly fewer exacerbations and required less intensive care for COPD or pneumonia in the 30 days following acute infection. Of note, the benefit of antiviral treatment was long lasting and improvements in healthcare outcomes and costs were sustained for at least 13 months after the index influenza episode.

Frequent and severe exacerbations are associated with higher rates of HRU and associated costs in patients with COPD.^4^, ^20^ Antiviral treatment was associated with a significantly lower risk of COPD exacerbation (10.4% in treated patients vs 18.2% in untreated patients) in the month after the index infection. Importantly, there was a sustained reduction in exacerbations in the antiviral‐treated group compared with the untreated group over the entire 13‐month period of the study. In addition to a reduction in exacerbations, treated patients also experienced a reduction in the frequency and number of inpatient, ED, and outpatient visits compared with untreated patients at all periods. Our previous study documented the longer‐term deleterious effects of influenza in patients with COPD and showed that the risk of exacerbations and HRU for COPD‐related complications remains high for at least 13 months after an acute influenza episode.^20^ The current study demonstrates that timely antiviral treatment of the index influenza episode provides a sustained benefit for at least 13 months postinfection.

Inpatient visits associated with acute exacerbations account for the largest share of direct medical costs due to COPD, and these costs increase with the severity of exacerbations.^28^ In the current study, antiviral treatment was associated with significantly lower COPD‐related costs in all HRU categories over all periods (the exception being ED visits during the first month). Inpatient care accounted for the largest share of direct medical costs during month 1; these costs were reduced substantially over the first month and remained lower over the full year in treated patients.

In addition to the direct effects of influenza virus on respiratory tissue, synergistic interactions between influenza virus and respiratory pathogens increase the risk for secondary bacterial infection.^29^ Pneumonia is a common complication of influenza, particularly in high‐risk patient groups such as patients with COPD, and approximately one‐third of hospitalized patients with laboratory‐confirmed influenza have pneumonia.^30^ COPD patients with exacerbations who develop pneumonia have worse outcomes in terms of mortality, hospitalization, readmission, and ventilator use than those without pneumonia.^31^ We show that among COPD patients diagnosed with influenza, those who received antiviral treatment had a significant reduction in HRU related to inpatient, ED, and outpatient visits for pneumonia not only in the postinfection month but also over the following full year. Costs in all HRU categories were reduced for treated patients; in particular, costs of inpatient visits, which was the largest cost driver of pneumonia‐related costs, were reduced significantly at all periods in treated patients. In similarity with its enhanced long‐lasting effect on COPD‐related HRU, influenza also has a sustained long‐term deleterious impact on pneumonia‐related HRU in patients with COPD,^20^ which was mitigated with antiviral treatment.

Despite expert recommendations to start antiviral treatment within 48 h of onset of symptoms in high‐risk patients with influenza, the use of antivirals to reduce influenza‐associated complications is suboptimal in this patient group.^21^, ^25^, ^32^, ^33^ Previous studies have documented the reduction in morbidity, mortality, and influenza‐associated complications in the general patient population and in at‐risk populations following antiviral treatment.^34^, ^35^, ^36^ This report shows that prompt antiviral use can improve healthcare outcomes and lower the cost impact of influenza‐associated respiratory complications over a prolonged period in patients with COPD.

The use of a large commercial claims database allowed for a large real‐world sample to be analyzed and for patients to be followed longitudinally to capture the long‐term effects of treatment. Also, treated and untreated cohorts were matched for baseline morbidity and severity of COPD as well as for demographic and other variables that could influence outcomes. However, the study has limitations. The results are based on claims data from a database that includes enrollees in commercial health plans and some supplemental Medicare plans and thus may not be transferable to the overall US population. The study was not randomized, and although propensity score matching was used to reduce selection bias, the possibility of residual bias could not be discounted. The influenza cases included here were identified by clinical diagnosis and were not necessarily confirmed by laboratory‐based influenza testing; cases with other viral illnesses may therefore have been included. Our analysis could not fully account for differences in healthcare‐seeking behaviors between the two cohorts. It is possible that patients in the antiviral‐treated cohort may have been more motivated to seek care and were also more likely to adhere to protective measures, which may have contributed to improved outcomes in this group. Additionally, influenza and pneumococcal vaccination status for patients was unavailable in the database used and thus was not included in the matching algorithm. Vaccination status could have impacted results if vaccination was unevenly distributed between the treated and untreated cohorts. Reporting of influenza vaccinations in claims databases is inconsistent and can be explained partially by patients receiving influenza vaccines outside physician's offices.^37^, ^38^ COPD exacerbations and pneumonia were identified from claims for office visits based on diagnosis codes in the claims databases and were not verified by review of medical records, which may have introduced errors because of missing information or inclusion of unconfirmed events. The impact of antiviral treatment on milder complications that can affect daily functioning but do not typically require medical attention could not be assessed because these were not recorded in the database. The inclusion of patients in the treatment group was based solely on a prescription claim; there was no verification that the patients actually took the medication.

## 5 CONCLUSIONS

In this study, using claims data for five influenza seasons (2012 through 2016), we demonstrate that antiviral treatment within 48 h of an influenza episode in patients with COPD resulted in significantly lower HRU and costs for respiratory complications not only over the first month after the influenza diagnosis but also over the full year after acute influenza infection. Patients who received an antiviral agent had fewer COPD exacerbations and COPD‐related hospitalizations, ED visits, and outpatient visits. Similarly, short‐ and long‐term HRU and costs of pneumonia‐related complications were also substantially lower in patients receiving an antiviral agent. Thus, this study of a large population of patients with COPD in a real‐world setting underscores that timely treatment of influenza in these patients provides long‐term clinical benefit and has a sustained and robust effect on lowering healthcare resources and costs.

## AUTHOR CONTRIBUTIONS

**Christopher Wallick:** Conceptualization; funding acquisition; investigation; project administration; supervision; visualization; writing—original draft; writing—review and editing. **Tu My To:** Data curation; formal analysis; methodology; project administration; resources; software; validation; writing—review and editing. **Stephan Korom:** Conceptualization; investigation; methodology; project administration; supervision; visualization; writing—original draft; writing—review and editing. **Henry Masters III:** Conceptualization; formal analysis; investigation; methodology; resources; supervision; visualization; writing—original draft; writing—review and editing. **Ning Wu:** Data curation; formal analysis; investigation; methodology; resources; software; validation; writing—review and editing. **Dalia Moawad:** Data curation; formal analysis; resources; software; validation; writing—review and editing. **Nicola A. Hanania:** Conceptualization; supervision; visualization; writing—review and editing.

## CONFLICT OF INTEREST STATEMENT

CW, TMT, SK, HM III, NW, and DM are current or past employees of Genentech/Roche, Inc. and hold Roche stock.

## ETHICS APPROVAL STATEMENT/PATIENT CONSENT STATEMENT

The study used deidentified data and was exempt from Institutional Review Board review. The research was compliant with the Health Insurance Portability and Accountability Act.

## PREVIOUS PRESENTATIONS

This study was presented at the Options X for the Control of Influenza conference, Singapore, August 28, 2019–September 1, 2019.

## Data Availability

The data that support the findings of this study are available from IBM® MarketScan® Research Databases, but these data are not publicly available. All relevant data are provided within the manuscript and supporting files.
